# Anti-Obesity Effects of *Rosa rugosa* Thunb. Flower Bud Extracts on Lipid Metabolism Regulation in 3T3-L1 Adipocytes and Sprague Dawley Rats

**DOI:** 10.3390/ijms26146963

**Published:** 2025-07-20

**Authors:** Jung Min Kim, Kyoung Kon Kim, Hye Rim Lee, Jae Cheon Im, Tae Woo Kim

**Affiliations:** Newgen Healthcare Co., Ltd., Chuncheon-si 24232, Republic of Korea; jmkim@newgenhc.co.kr (J.M.K.); gon87@newgenhc.co.kr (K.K.K.); hyerim0708@newgenhc.co.kr (H.R.L.); ccyim@newgenhc.co.kr (J.C.I.)

**Keywords:** obesity, lipid metabolism, high-fat diet, *Rosa rugosa* Thunb. flower bud, NG-RR-T1F

## Abstract

In modern society, obesity and its associated complications have emerged as serious public health concerns, primarily stemming from sedentary lifestyles and carbohydrate-rich diets. Due to the severe side effects often associated with pharmacological anti-obesity agents, emerging global efforts focus on preventive strategies, e.g., dietary modifications and weight gain-suppressing functional foods. In this context, plant-derived metabolites are extensively investigated for their beneficial anti-obesity effects. In this study, we evaluated how *Rosa rugosa* Thunb. flower bud extract affects fat metabolism in 3T3-L1 preadipocyte cells. The extract significantly inhibited adipocyte differentiation and intracellular triglyceride accumulation in 3T3-L1 cells, enhanced lipolysis, suppressed lipogenesis, and promoted energy metabolism in differentiated adipocytes. In vivo, it reduced body and organ weights and fat mass in high-fat diet-induced obese rats, along with marked adipocyte size and hepatic lipid accumulation reductions. In the epididymal adipose tissue, the extract similarly enhanced lipolytic activity, suppressed lipogenic enzyme expression, and stimulated energy expenditure. Taken together, our results demonstrate the potential of *R. rugosa* Thunb. flower bud extract in reducing fat accumulation through lipid metabolism modulation both in cellular and animal models. Further studies are warranted to identify the active constituents and evaluate the safety and efficacy of the extract in clinical applications.

## 1. Introduction

The global prevalence of obesity has markedly increased, driven by aging societies and widespread lifestyle modifications, thereby representing substantial public health challenges and economic burdens [1,2]. Obesity is a multifactorial metabolic disorder driven by dysregulated energy homeostasis—primarily a sustained imbalance between caloric intake and expenditure—shaped by genetic, environmental, and lifestyle factors. In the Republic of Korea, obesity prevalence escalated from 32.6% in 2009 to 38.5% in 2018, corresponding to a 1.18-fold increase [3]. In the United States, adult obesity prevalence was approximately 42.4% in 2017–2018 [4]. According to projections, by 2030, the national prevalence of adult obesity is expected to reach 48.9%, with 24.2% classified as severely obese; in 29 states, more than half of the adult population is projected to be obese [5]. Furthermore, recent modeling estimates indicate that over 58% of U.S. adults will be obese by 2050, reflecting a steadily escalating public health burden [6]. Globally, adult obesity rates increased from 28.8% to 36.9% in men and from 29.8% to 38.0% in women between 1980 and 2013, with particularly high rates reported in countries such as Germany, the United Kingdom, Italy, and the United States [2]. In addition, the obesity-associated socioeconomic burden in the Republic of Korea was estimated at USD 9.665 billion in 2016 and USD 21.49 billion in 2019, comprising both direct and indirect costs [7,8,9]. Projections suggest that this burden could increase dramatically, reaching approximately USD 46 billion by 2030 [9]. In the U.S., annual medical costs per obese individual reached USD 1901 in 2014, contributing to national expenses of USD 149.4 billion [10,11].

Obesity is tightly linked to insulin resistance, lipotoxicity, and adipocyte dysfunction, which elevate circulating FFAs, ROS, and proinflammatory adipokines, factors that drive metabolic disorders such as type 2 diabetes and dyslipidemia [12,13,14]. Thus, improving lipid metabolism represents a crucial therapeutic strategy for obesity management.

The 5′-adenosine monophosphate-activated protein kinase (AMPK) signaling pathway is a central cellular energy homeostasis regulator pathway [15]. AMPK activation inhibits lipogenesis by downregulating key transcriptional regulators, including peroxisome proliferator-activated receptor gamma (PPARγ), CCAAT/enhancer-binding protein alpha (C/EBPα), and sterol regulatory element binding protein 1c (SREBP-1c). Fatty acids (FAs), stored as triacylglycerols within lipid droplets (LDs), are hydrolyzed into FAs and glycerol through lipolysis upon energy demand; sequentially catalyzed by adipose triglyceride lipase (ATGL), hormone-sensitive lipase (HSL), and monoglyceride lipase (MGL); and tightly regulated by signaling protein (e.g., protein kinase A (PKA), alcohol dehydrogenase 5, and perilipin 1A) phosphorylation [16,17]. Concurrently, intracellular acetyl-CoA is converted into malonyl-CoA, then to palmitate via acetyl-CoA carboxylase (ACC)- and fatty acid synthase (FAS)-related enzymatic effects, contributing to triglyceride synthesis. Therefore, modulating lipogenesis-, lipolysis-, and energy expenditure-related pathways is essential for reducing intracellular triglyceride accumulation, potentially offering a therapeutic avenue for preventing metabolic disorders and associated diseases.

Conventional treatments include diet, exercise, and behavioral therapy but often require pharmacologic or surgical intervention when these fail [18,19]. The anti-obesity drug market is expanding at the annual rate of 24–27%, projected to grow from USD 24 billion in 2023 to USD 74 billion by 2028 [9,20]. However, common medications may cause side effects such as depression, hepatotoxicity, or dependence [21,22]. In response, interest in functional foods has grown. These scientifically validated products—referred to as “health functional foods”, “dietary supplements”, or “food supplements” depending on the region—offer lower side-effect profiles. In the Republic of Korea, the functional food market reached KRW 3.3 trillion in 2020, with body fat-reducing ingredients like *Garcinia cambogia*, *Camellia sinensis* (Pu-erh tea), and Cissus quadrangularis drawing consumer attention [23]. These ingredients show anti-obesity effects in vitro and in vivo, and some have been approved based on clinical trials.

*R. rugosa* Thunb., a deciduous shrub of the Rosaceae family, has been traditionally used in East Asian (e.g., Korean, Chinese, and Japanese) medicine. The plant contains a range of bioactive compounds such as anthocyanins (e.g., cyanidins, pelargonidins, and peonidins), flavonoids (e.g., kaempferol and quercetin), and phenolic acids (e.g., gallic, ellagic, and quinic acids) [24,25]. Various parts of the plant, including its roots, stems, seeds, and fruit pulp, reportedly retain antihyperlipidemic, antiallergic, anti-inflammatory, sedative, whitening, anti-aging, and antidiabetic properties [24,26]. Plant development and maturation involve biochemical and physiological regulation via gene expression and enzymatic activity [27]. Maturity-dependent differences have been observed in the antioxidant activities of blackberries, strawberries, and red raspberries as well as in the ascorbic acid content in paprika across different developmental stages [28].

In this study, we analyzed the marker compounds in *R. rugosa* Thunb. flower bud extracts collected at different blooming stages and assessed how they affect lipid accumulation. Among the extracts, the sample exhibiting the strongest inhibitory effect on lipid accumulation was further evaluated for its impact on lipolysis, lipogenesis, and energy metabolism in 3T3-L1 adipocytes and rats. Our results provide preliminary evidence supporting this extract as a potent candidate for body fat-reducing functional health food development.

## 2. Results

### 2.1. R. rugosa Thunb. Flower Bud Extract Marker Compound Analysis

**Figure 1:**
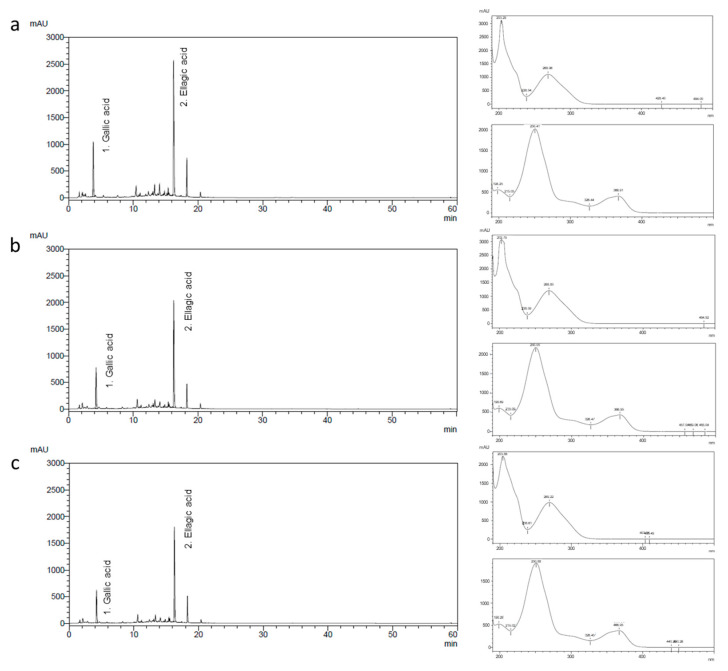
Chromatograms and UV spectra of gallic acid (1) and ellagic acid (2) detected in the *R. rugosa* Thunb. flower bud extract (**a**) 3, (**b**) 6, and (**c**) 9 days after flower bud blooming.

We analyzed the marker compounds in *Rosa rugosa* Thunb. flower bud extracts at different blooming stages using high-performance liquid chromatography (HPLC) and identified gallic acid and ellagic acid as key constituents. Notably, the peak intensities of both compounds decreased progressively with advancing bloom stage (Figure 1). The gallic acid contents on days 3, 6, and 9 were 44.19 ± 0.87 mg/g, 30.25 ± 0.76 mg/g, and 21.42 ± 0.42 mg/g, respectively, while the ellagic acid contents were 33.39 ± 0.32 mg/g, 20.25 ± 0.21 mg/g, and 14.14 ± 0.59 mg/g, respectively.

### 2.2. Blooming Stage-Related R. rugosa Thunb. Flower Bud Extract Cytotoxicity in 3T3-L1 Preadipocyte Cells

**Figure 2:**
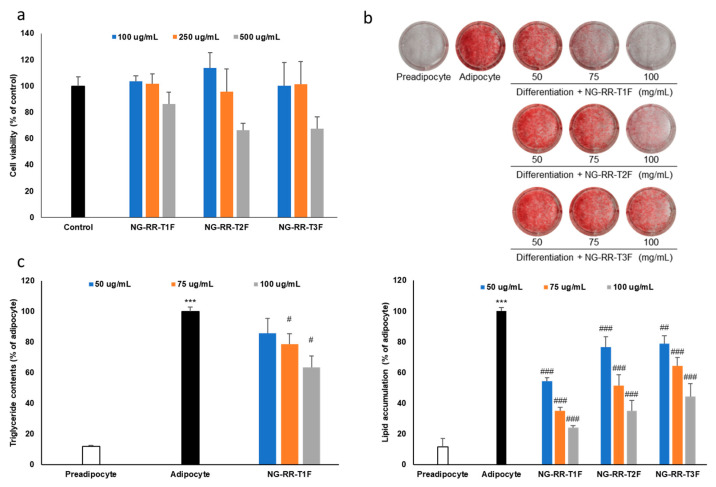
Effects of *R. rugosa* Thunb. flower bud extracts on fat accumulation in 3T3-L1 adipocytes. The cells were differentiated into adipocytes in the presence or absence of *R. rugosa* Thunb. flower bud extracts. (**a**) Cell viability assay of *R. rugosa* Thunb. flower bud extracts in 3T3-L1 preadipocyte cells. (**b**) Quantification of intracellular lipid content accumulation. (**c**) Intracellular triglyceride content quantification. The values are expressed as the mean ± standard deviation (SD). The white bar indicates the preadipocyte group, and the black bar represents the adipocyte group without treatment. The blue, orange, and gray bars correspond to the treatment groups with *R. rugosa* Thunb. flower bud extracts. Each data point represents the mean value of samples in triplicate. *** *p* < 0.001 vs. preadipocyte cells; # *p* < 0.05, ## *p* < 0.01, and ### *p* < 0.001 vs. the adipocyte group.

Prior to evaluating the anti-obesity effects, we assessed the cytotoxicity of the flower bud extracts in 3T3-L1 preadipocyte cells using the 3-(4,5-dimethylthiazol-2-yl)-2,5-diphenyl tetrazolium bromide (MTT) assay. At concentrations of 0.1 and 0.25 mg/mL, all treatments yielded cell viabilities above 80%, indicating no significant cytotoxicity. However, at 0.5 mg/mL, cell viability dropped below 80%, suggesting cytotoxic effects at higher concentrations (Figure 2a). Consequently, we conducted subsequent experiments at maximum concentrations of 0.25 mg/mL to avoid cytotoxic interference.

### 2.3. Lipid Accumulation Inhibitory Effects of R. rugosa Thunb. Flower Bud Extracts During Adipocyte Differentiation

To assess how the flower bud extracts affect lipid accumulation, we induced 3T3-L1 preadipocyte cells to differentiate into adipocytes in the presence of extracts at various blooming stages. The extract from day 3 buds (NG-RR-T1F) displayed the strongest lipid accumulation inhibitory effect, followed by day 6 and 9 bud extracts (NG-RR-T2F and NG-RR-T3F, respectively), indicating an inverse correlation between bloom maturity and efficacy (Figure 2b). Therefore, due to its superior lipid-lowering potential, we selected NG-RR-T1F for further evaluation.

### 2.4. NG-RR-T1F Extract Suppresses Intracellular Triglyceride Formation

To evaluate how NG-RR-T1F affects TG accumulation during adipocyte differentiation, we treated 3T3-L1 cells with the extract throughout the entire cell differentiation period. NG-RR-T1F treatment significantly reduced intracellular TG levels without affecting cell viability, confirming its anti-adipogenic effect (Figure 2c).

### 2.5. NG-RR-T1F Regulates Lipolysis-Related mRNA and Protein Expressions

**Figure 3:**
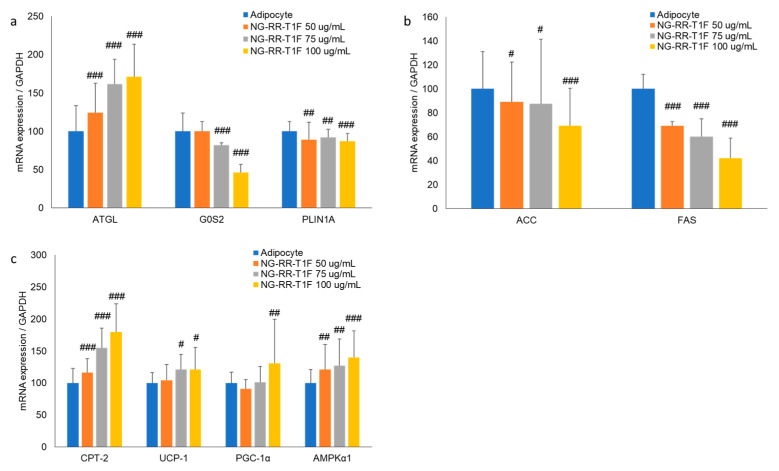
Effects of NG-RR-T1F on lipid metabolism-related mRNA expression in 3T3-L1 adipocytes. Cells were differentiated into adipocytes in the presence or absence of NG-RR-T1F. (**a**) NG-RR-T1F regulates lipolysis-related mRNA expression. (**b**) NG-RR-T1F regulates lipogenesis-related mRNA expression. (**c**) NG-RR-T1F enhances energy expenditure-related mRNA expression. # *p* < 0.05, ## *p* < 0.01, and ### *p* < 0.001 vs. the adipocyte group.

**Figure 4:**
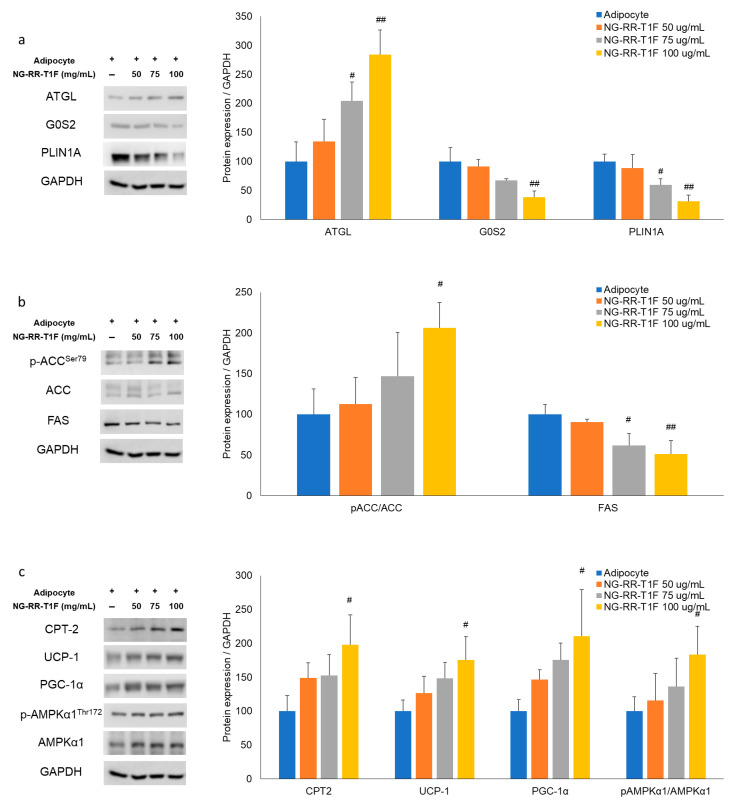
Effects of NG-RR-T1F on lipid metabolism-related protein expression in 3T3-L1 adipocytes. Cells were differentiated into adipocytes in the presence or absence of NG-RR-T1F. (**a**) NG-RR-T1F regulates lipolysis-related protein expression. (**b**) NG-RR-T1F regulates lipogenesis-related protein expression. (**c**) NG-RR-T1F enhances energy expenditure-related protein expression. # *p* < 0.05 and ## *p* < 0.01 vs. the adipocyte group.

Next, we performed RT-qPCR Western blot analysis and demonstrated that NG-RR-T1F modulated lipolysis-related mRNA and protein expressions in differentiated 3T3-L1 adipocytes. Specifically, ATGL mRNA and protein expression concentration-dependently increased, while that of perilipin and G0S2 decreased (Figure 3a and Figure 4a). At 50, 75, and 100 µg/mL NG-RR-T1F concentrations, ATGL mRNA expression increased to 124%, 162%, and 172%, and protein levels rose to 134%, 204%, and 284% relative to the control adipocytes, respectively. Conversely, G0S2 mRNA and protein levels decreased to 100%, 82%, and 47% and to 91%, 68%, and 39%, respectively. Perilipin expression also declined to 89%, 92%, and 87% at the mRNA level and to 89%, 60%, and 32% at the protein level compared with adipocyte values.

### 2.6. NG-RR-T1F Regulates Lipogenesis-Related mRNA and Protein Expression

To determine how NG-RR-T1F affects lipogenesis, we assessed phosphorylated ACC and FAS mRNA and protein expressions (Figure 3b and Figure 4b). NG-RR-T1F treatment led to a decrease in ACC mRNA expression and an increase in ACC phosphorylation, while both FAS mRNA and protein levels were reduced in a concentration-dependent manner. Specifically, ACC mRNA expression decreased to 89%, 88%, and 69%, while p-ACC protein levels increased to 112%, 147%, and 206%, at 50, 75, and 100 µg/mL, respectively. FAS mRNA levels were reduced to 69%, 60%, and 42%, and FAS protein levels decreased to 91%, 62%, and 51% compared to control adipocytes.

### 2.7. NG-RR-T1F Enhances Energy Expenditure-Related mRNA and Protein Expression

NG-RR-T1F also promoted energy expenditure, as evidenced by increased CPT-2, UCP1, PGC-1α, and phosphorylated AMPK expressions (Figure 3c and Figure 4c). Treatment with NG-RR-T1F at concentrations of 50, 75, and 100 µg/mL increased the mRNA expression levels of CPT-2 to 116%, 155%, and 180%; UCP-1 to 104%, 121%, and 121%; PGC-1α to 91%, 101%, and 131%; and AMPK to 121%, 127%, and 140%, respectively, compared to the control. Similarly, the protein expression levels of CPT-2 increased to 149%, 153%, and 198%; UCP-1 to 127%, 148%, and 175%; PGC-1α to 146%, 176%, and 210%; and phosphorylated AMPK to 116%, 136%, and 184%, respectively.

### 2.8. NG-RR-T1F Reduces Body Weight in High-Fat Diet (HFD)-Induced Obese Rats

**Figure 5:**
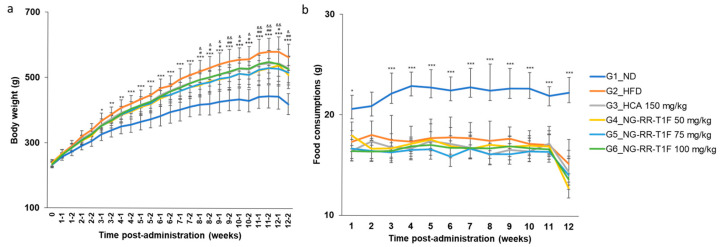
Effects of NG-RR-T1F on body weight and food consumption in HFD-induced obese rats after 12 weeks. (**a**) Body weight gain. (**b**) Food consumption. HFD: high-fat diet; HCA: hydroxycitric acid; NG-RR-T1F: *R. rugosa* Thunb. flower bud extracts. The values are expressed as the mean ± SD (*n* = 8). * *p* < 0.05, ** *p* < 0.01, and *** *p* < 0.001 vs. G1_ND; # *p* < 0.05 and ## *p* < 0.01 vs. G2_HFD (G4 vs. G2); & *p* < 0.05 and && *p* < 0.01 vs. G2_HFD (G5 vs. G2).

Significant differences emerged in body weight between the normal diet (ND) and HFD groups (G1 and G2, respectively) by week 3. NG-RR-T1F administration at doses of 50 mg/kg (G4) and 75 mg/kg (G5) resulted in significant body weight reduction beginning week 8 compared to the HFD group, and this effect persisted until the end of the study. Although a dose of 100 mg/kg (G6) also reduced body weight, the difference was not statistically significant. At the end of the study, the mean body weights in G2 and G4 were 564.31 and 509.43 g, representing a 9.72% reduction in the latter. G5 and G6 exhibited average mean weights of 517.49 and 527.29 g, corresponding to 8.30% and 6.56% reductions, respectively. We observed no significant differences in food intake among the groups, suggesting that the observed weight loss was not attributable to reduced appetite (Figure 5b).

### 2.9. NG-RR-T1F Reduces Organ and Fat Depot Weights in HFD-Induced Rats

**Figure 6:**
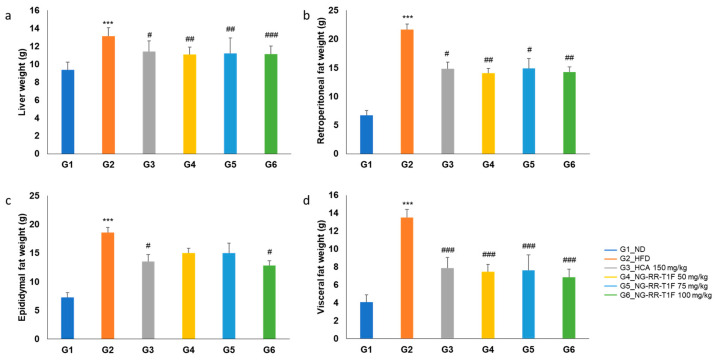
Effects of NG-RR-T1F on absolute organ weights in HFD-induced obese rats. (**a**) Liver weight, (**b**) retroperitoneal fat weight, (**c**) epididymal fat weight, and (**d**) visceral fat weight. The values are expressed as the mean ± SD (*n* = 8). *** *p* < 0.001 vs. G1_ND; # *p* < 0.05, ## *p* < 0.01, and ### *p* < 0.001 vs. G2_HFD.

Liver weight significantly increased in G2 (13.11 g) compared to G1 (9.38 g). NG-RR-T1F administration significantly reduced liver weight in all treatment groups as follows: G4 (11.07 g), G5 (11.19 g), and G6 (11.47 g) (Figure 6a). Retroperitoneal fat weight increased significantly in G2 (21.67 g vs. 6.71 g in G1) but significantly decreased in G4 (14.07 g), G5 (15.80 g), and G6 (16.05 g) (Figure 6b). Moreover, epididymal fat weight significantly increased in the HFD group (18.55 vs. 7.28 g in G1). Although we observed reductions in the NG-RR-T1F groups, i.e., G4 (15.02 g), G5 (15.01 g), and G6 (15.47 g), only the G6 group showed a statistically significant decrease, whereas G4 and G5 did not exhibit significant differences (Figure 6c). Visceral fat weight significantly increased in G2 (13.04 vs. 4.06 g in G1) and significantly decreased in G4 (7.46 g), G5 (8.43 g), and G6 (8.60 g) (Figure 6d).

### 2.10. NG-RR-T1F Improves Body Composition

**Figure 7:**
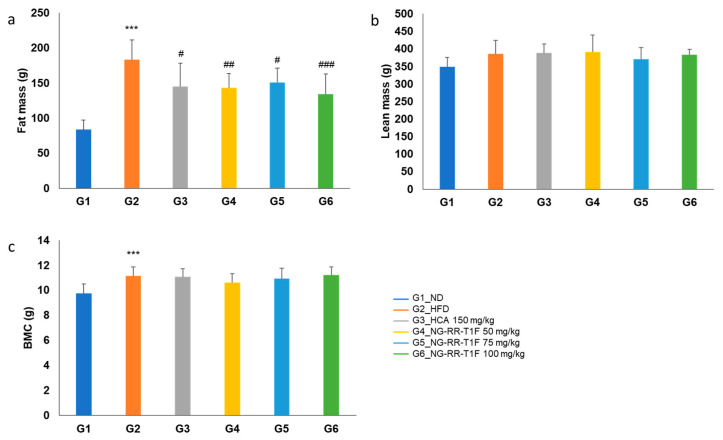
Effects of NG-RR-T1F on body compositions in HFD-induced obese rats. (**a**) Fat mass, (**b**) lean mass, and (**c**) bone mineral content. The values are expressed as the mean ± SD (*n* = 8). *** *p* < 0.001 vs. G1_ND; # *p* < 0.05 and ## *p* < 0.01, and ### *p* < 0.001 vs. G2_HFD.

Fat mass was significantly higher in the HFD (183.27 g) compared to the ND (84.02 g) group. NG-RR-T1F treatment significantly reduced fat mass to 138.33 g (G4), 146.62 g (G5), and 148.91 g (G6) (Figure 7a). Lean mass did not significantly differ among the groups: G2 (386.69 g), G1 (349.30 g), G4 (390.64 g), G5 (370.72 g), and G6 (383.04 g) (Figure 7b). Bone mineral content slightly increased in G2 (11.15 g) compared to G1 (9.77 g), but NG-RR-T1F exhibited no significant effect: G4 (10.63 g), G5 (10.95 g), and G6 (11.22 g) (Figure 7c).

### 2.11. NG-RR-T1F Modulates Serum Lipid Profiles

**Figure 8:**
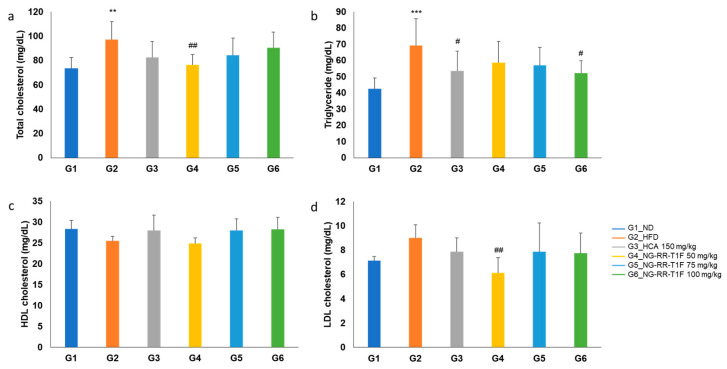
Effects of NG-RR-T1F on serum lipid parameter in HFD-induced obese rats. (**a**) Total cholesterol, (**b**) triglyceride, (**c**) HDL-C, and (**d**) LDL-C. The values are expressed as the mean ± SD (*n* = 8). ** *p* < 0.01 and *** *p* < 0.001 vs. G1_ND; # *p* < 0.05 and ## *p* < 0.01 vs. G2_HFD.

Serum triglyceride levels significantly increased in the HFD group (69.25 vs. 42.63 mg/dL in G1). Among the treatment groups, only G6 (52.13 mg/dL) yielded a statistically significant reduction compared to G2 (Figure 8a). Moreover, total cholesterol was significantly elevated in G2 (97.25 mg/dL vs. 73.25 mg/dL in G1). G4 (76.50 mg/dL) displayed a significant reduction compared to G2, while G5 (84.25 mg/dL) and G6 (90.38 mg/dL) did not (Figure 8b). Low-density lipoprotein cholesterol (LDL-C) levels were not significantly different between G2 and G1 (9.00 and 7.13 mg/dL, respectively). However, G4 (6.13 mg/dL) exhibited a significant reduction compared to G2 (Figure 8c). High-density lipoprotein cholesterol (HDL-C) levels did not differ significantly between G2 (25.50 mg/dL) and G1 (28.38 mg/dL), nor among NG-RR-T1F-treated groups (G4, 5, and 6: 24.88, 28.00, and 28.25 mg/dL, respectively) (Figure 8d).

### 2.12. NG-RR-T1F Improves Liver Function Markers

**Figure 9:**
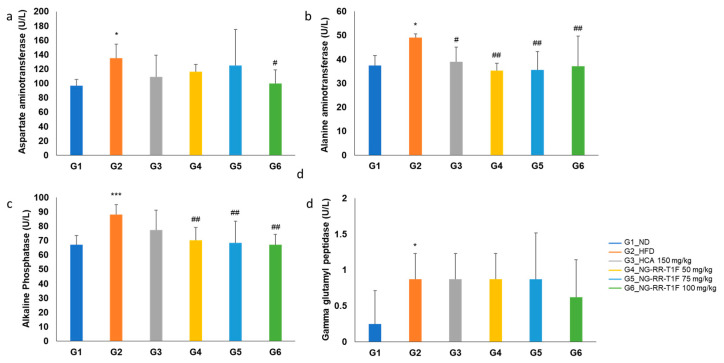
Effects of NG-RR-T1F on serum liver function parameters in HFD-induced obese rats. (**a**) AST, (**b**) ALT, (**c**) ALP, and (**d**) GGT. The values are expressed as the mean ± SD (*n* = 8). * *p* < 0.05 and *** *p* < 0.001 vs. G1_ND; # *p* < 0.05 and ## *p* < 0.01 vs. G2_HFD.

Serum aspartate aminotransferase (AST) levels were significantly higher in G2 than in G1 (135.13 and 96.88 U/L, respectively). G6 (100.00 U/L) exhibited a significant reduction compared to G2, while G4 (116.13 U/L) and G5 (124.75 U/L) did not (Figure 9a). Alanine aminotransferase (ALT) levels significantly increased in G2 (49.13 U/L vs. 37.50 U/L in G1) but significantly decreased in all the NG-RR-T1F groups, i.e., G4, 5, and 6 (35.25, 35.63, and 37.13 U/L, respectively) (Figure 9b). Alkaline phosphatase (ALP) levels were significantly higher in G2 (88.00 U/L vs. 67.00 U/L in G1) and significantly reduced in G4, 5, and 6 (70.25, 68.38, and 67.25 U/L, respectively) (Figure 9c). Gamma-glutamyltranspeptidase (GGT) levels were also elevated in G2 compared to G1 (0.88 and 0.25 U/L, respectively) and significantly reduced in G6 (0.63 U/L) but not in G4 and G5 (Figure 9d).

**Figure 10:**
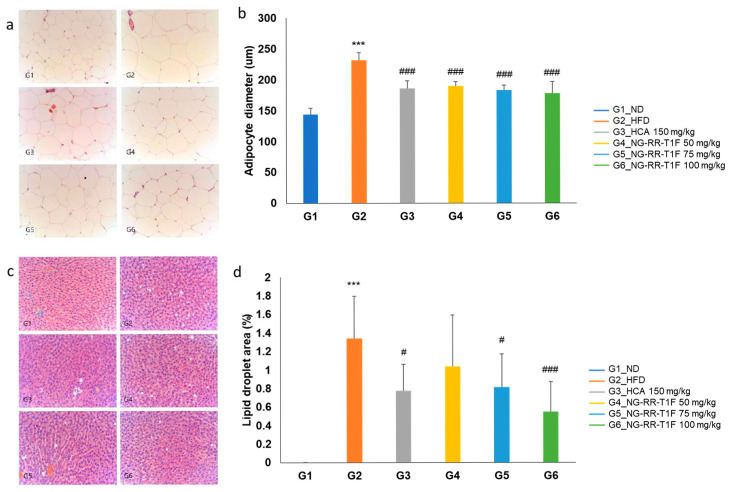
Effects of NG-RR-T1F on histopathological analysis in HFD-induced obese rat. (**a**,**b**) Retroperitoneal fat size and (**c**,**d**) hepatic LD area. The values are expressed as the mean ± SD (*n* = 8). *** *p* < 0.001 vs. G1_ND; # *p* < 0.05 and ### *p* < 0.001 vs. G2_HFD.

G1 (0%). NG-RR-T1F treatment significantly reduced the LD area in G5 (0.81%) and G6 (0.55%), with a non-significant reduction in G4 (1.04%) (Figure 10c,d).

### 2.13. NG-RR-T1F Reduces Hepatic Steatosis and Adipocyte Size

The average adipocyte diameter in the retroperitoneal fat tissue was significantly higher in G2 (231.59 µm) than in G1 (143.83 µm). All NG-RR-T1F groups comprised significantly smaller adipocytes, i.e., G4, 5, and 6 (189.13, 182.56, and 177.89 µm, respectively) (Figure 10a,b). The hepatic LD area was significantly increased in G2 (1.34%) compared to G1 (0%).

### 2.14. NG-RR-T1F Regulates Lipolysis, Lipogenesis, and Energy Expenditure-Related mRNA Protein Expression in the Epididymal Adipose Tissue of HFD Rats

**Figure 11:**
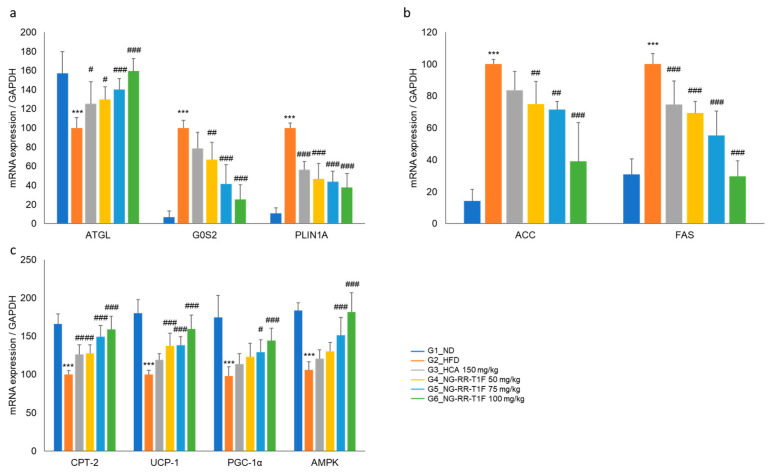
Effects of NG-RR-T1F on lipid metabolism-related mRNA expression in the epididymal fat tissue of HFD-induced obese rats. (**a**) NG-RR-T1F regulates lipolysis-related mRNA expression. (**b**) NG-RR-T1F regulates lipogenesis-related mRNA expression. (**c**) NG-RR-T1F enhances energy expenditure-related mRNA expression. The values are expressed as the mean ± SD (*n* = 8). *** *p* < 0.001 vs. G1_ND; # *p* < 0.05, ## *p* < 0.01 and ### *p* < 0.001 vs. G2_HFD.

**Figure 12:**
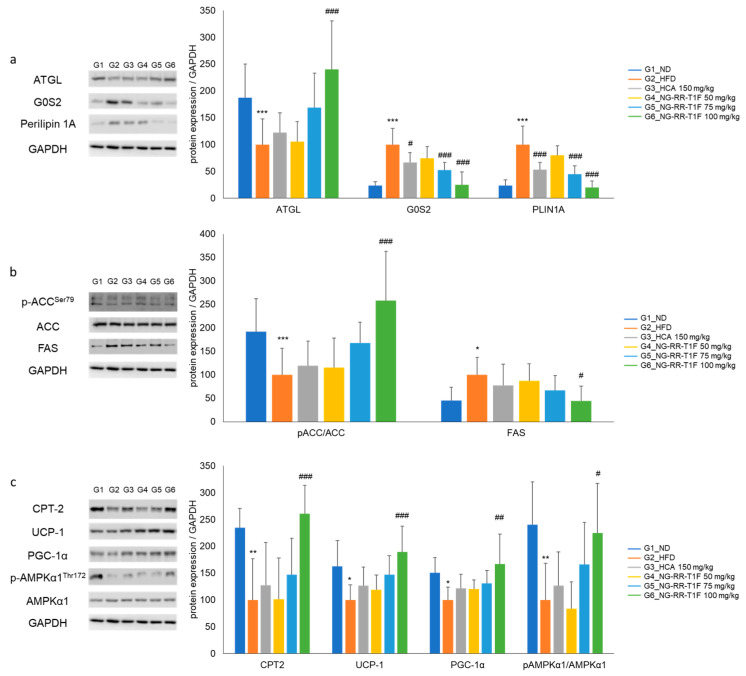
Effects of NG-RR-T1F on lipid metabolism-related protein expression in the epididymal fat tissue of HFD-induced obese rats. (**a**) NG-RR-T1F regulates lipolysis-related protein expression. (**b**) NG-RR-T1F regulates lipogenesis-related protein expression. (**c**) NG-RR-T1F enhances energy expenditure-related protein expression. The values are expressed as the mean ± SD (*n* = 8). * *p* < 0.05, ** *p* < 0.01, and *** *p* < 0.001 vs. G1_ND; # *p* < 0.05, ## *p* < 0.01, and ### *p* < 0.001 vs. G2_HFD.

To elucidate the underlying molecular mechanisms of the NG-RR-T1F-related anti-obesity effects in vivo, we examined lipolysis-, lipogenesis-, and energy metabolism-involved key mRNA and protein expressions in the epididymal adipose tissue of HFD-induced obese rats. RT-qPCR and Western blot analysis revealed that the in vivo expression patterns mirrored those observed in 3T3-L1 adipocytes (Figure 11 and Figure 12). Specifically, the NG-RR-T1F treatment enhanced ATGL expression, AMPK phosphorylation, CPT-2, UCP-1, and PGC-1 α levels. In contrast, FAS and G0S2 expressions significantly decreased.

## 3. Discussion

AMPK, a key cellular energy sensor, is central to regulating energy homeostasis and is reportedly a critical inhibitory regulator of adipocyte differentiation and obesity progression [29]. Pharmacological AMPK activators (e.g., AICAR and various naturally derived compounds) reportedly suppress adipogenesis by transcription factor (e.g., PPARγ, C/EBPα, and FAS) downregulation. These compounds also promote fat metabolism by enhancing lipase activity within LDs, thereby promoting triglyceride hydrolysis into free fatty acids [29,30,31].

In this study, we demonstrated that NG-RR-T1F, an *R. rugosa* Thunb. flower bud-derived hot water extract, modulates lipid metabolism by regulating lipolysis, lipogenesis, and energy expenditure both in 3T3-L1 adipocytes and HFD-induced obese rats. The enzymatic cascade ATGL, HSL, and MGL mediates lipolysis, which is further regulated by cofactors including comparative gene identification-58 (CGI-58), perilipin 1A, and G0/G1 Switch 2 (G0S2) [32]. Among these, ATGL is activated through its interaction with CGI-58, the latter of which is released from its complex with perilipin 1A upon upstream signals (e.g., PKA and AMPK phosphorylation) [33]. Moreover, AMPK reportedly enhances ATGL-mediated lipolytic activity directly, further supporting its role in lipid catabolism regulation. Lipogenesis is a metabolic process comprising acetyl-CoA conversion into malonyl-CoA and palmitate through the enzymatic activities of ACC and FAS, ultimately resulting in triglyceride synthesis and storage. Excessively activated lipogenesis contributes to abnormal triglyceride accumulation and dysregulated adipokine secretion, hallmark features of obesity. Under certain conditions, surplus acetyl-CoA could be redirected toward mitochondrial β-oxidation through enzymatic activities such as those of acyl-CoA synthetase and CPT, functioning as compensatory mechanisms for energy regulation. CPT-2 (carnitine palmitoyltransferase-2) is essential for transporting long-chain fatty acids into the mitochondrial matrix, enabling their subsequent β-oxidation [34]. UCP-1 (uncoupling protein-1), mainly expressed in brown and beige adipocytes, dissipates the mitochondrial proton gradient to generate heat, mediating non-shivering thermogenesis and enhanced energy expenditure [35]. PGC-1α (peroxisome proliferator-activated receptor gamma coactivator-1 alpha) is a master regulator of mitochondrial biogenesis and oxidative metabolism, promoting enhanced mitochondrial capacity and thermogenic gene expression [36]. AMPK acts as a central metabolic switch, promoting catabolic processes by β-oxidation enhancement. In our study, NG-RR-T1F increased AMPK phosphorylation, downregulated lipogenic enzymes, and stimulated lipolysis and energy expenditure, collectively supporting its anti-adipogenic and lipid-lowering effects.

Obesity results from a chronic imbalance between energy intake and expenditure, leading to excess energy storage in the form of triglycerides in the adipose tissue [37]. To evaluate the anti-obesity potential of NG-RR-T1F, we used an HFD-induced obese rat model, which reliably exhibits hallmark obesity features, including increased fat mass, adipocyte hypertrophy, and dyslipidemia [38]. Upon 1-week NG-RR-T1F administration at doses of 50, 75, and 100 mg/kg, we observed body weight gain reduction without changes in food intake, indicating that the extract facilitates appetite suppression-independent fat loss. In line with previous studies, NG-RR-T1F significantly lowered triglyceride, total cholesterol, and LDL-C serum levels, thereby alleviating HFD-induced hyperlipidemia and dyslipidemia [39].

To further elucidate the underlying mechanisms, we examined metabolic protein expressions in the epididymal adipose tissue of HFD-induced obese rats. Our in vivo and in vitro results were consistent, demonstrating that NG-RR-T1F upregulates ATGL expression and enhances AMPK phosphorylation while concurrently downregulating lipogenic enzyme (e.g., ACC and FAS) expression. These changes indicate coordinated adipogenesis, lipolysis, and overall energy metabolism regulation. Adipogenesis is a multistep process involving preadipocyte cells proliferation, differentiation, fatty acid synthesis, and lipid storage, all governed by tightly regulated molecular signaling networks [40]. AMPK functions as a central metabolic switch, inhibiting anabolic pathways (including lipo- and adipogenesis) while promoting catabolic processes (e.g., fatty acid oxidation). Furthermore, AMPK reportedly suppresses PPARγ signaling, thereby hindering preadipocyte differentiation into mature adipocytes and contributing to adipose tissue expansion inhibition [10]. LDs, previously considered passive energy reservoirs, are now recognized as dynamic organelles that participate in various cellular processes. ATGL not only mobilizes fatty acids from LDs but also activates key metabolic signaling pathways, including PPARα and its coactivator PPARγ coactivator 1 alpha (PGC-1α), thereby promoting mitochondrial biogenesis and enhancing fatty acid oxidation [41]. Emerging evidence further indicates that ATGL-mediated lipolysis stimulates sirtuin 1 activity, subsequently amplifying PPARα/PGC-1α signaling, thereby highlighting the intricate interplay between lipid mobilization and oxidative metabolism [41]. In summary, these discoveries underscore the pivotal role of ATGL in coupling intracellular lipid turnover with systemic energy homeostasis and metabolic health.

**Figure 13:**
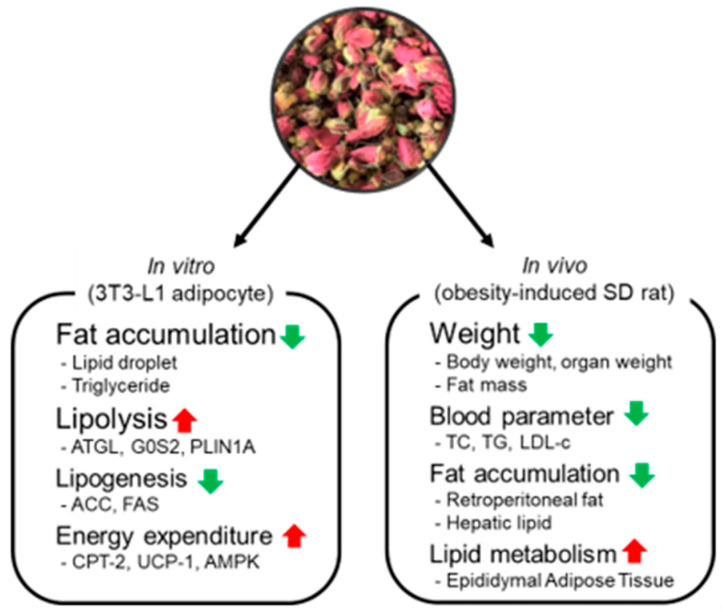
Effects of NG-RR-T1F on lipid metabolism in 3T3-L1 adipocyte and SD rat with high-fat diet-induced obesity. Red arrows indicate upregulation, and green arrows indicate downregulation of the respective biomarkers or outcomes.

Taken together, our results demonstrate that NG-RR-T1F modulates lipid metabolism by lipolysis enhancement, lipogenesis inhibition, and mitochondrial energy expenditure promotion both in cellular and living animal models (Figure 13). If the safety and efficacy of NG-RR-T1F are confirmed in future clinical studies, this extract could serve as a promising candidate for functional food development aimed at obesity management. Furthermore, compared to other commercially available fat-reducing ingredients, NG-RR-T1F—derived from domestically sourced *Rosa rugosa* flower buds—offers advantages in terms of production scalability, cost efficiency, and ease of standardization, as it can be cultivated, extracted, and managed entirely within the Republic of Korea. These factors collectively support its potential competitiveness in the functional food market.

## 4. Materials and Methods

### 4.1. R. rugosa Thunb. Flower Bud Hot-Water Extract Preparation

**Figure 14:**
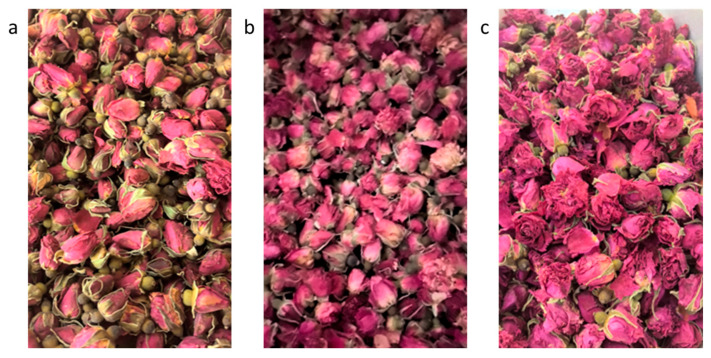
Flowering degree phenotype of *R. rugosa* Thunb. flower buds (**a**) 3, (**b**) 6, and (**c**) 9 days after flower bud blooming.

We procured *R. rugosa* Thunb. flower buds collected from Shandong Province, China, and categorized them based on their blooming stage, i.e., days 3, 6, and 9 (NG-RR-T1F, NG-RR-T2F, and NG-RR-T3F, respectively) (Figure 14). Each sample was extracted in duplicate from dried flower buds using 15 volumes (*w*/*v*) of distilled water at 100 °C for 4 h; then, we filtered the extracts through a 1 µm filter, concentrated them to 20° Brix, and subsequently freeze-dried them.

### 4.2. HPLC Analysis of R. rugosa Thunb. Flower Bud Extracts

To quantify phenolic acids in the flower bud extracts, we performed HPLC using an LC-20A system (Shimadzu, Kyoto, Japan) equipped with a photodiode array detector (SPD-M20A, Shimadzu, Kyoto, Japan) and a ZORBAX Eclipse XDB-C18 column (150 mm × 4.6 mm, 3.5 µm, Agilent Technologies, Santa Clara, CA, USA). The mobile phase consisted of solvents A (0.2% formic acid in distilled water) and B (0.2% formic acid in acetonitrile) at a flow rate of 1 mL/min. We maintained the column temperature at 30 °C and set the detection wavelength to 254 nm, applying a gradient elution, and injecting 10 µL of each sample under the conditions described in Table 1. We used gallic (99%, Aladdin, Shanghai, China) and ellagic (>98%, Aladdin, Shanghai, China) acids as standards. We prepared stock solutions of 1000 µg/mL and diluted them with 20% methanol prior to analysis.

### 4.3. Cell Culture and Differentiation

We obtained 3T3-L1 preadipocyte cells from the American Type Culture Collection (Manassas, VA, USA) and cultured them in Dulbecco’s Modified Eagle Medium (DMEM; Welgene, Daegu, Republic of Korea) supplemented with 10% bovine calf serum (Welgene, Daegu, Republic of Korea) and 1% penicillin–streptomycin (Welgene, Daegu, Republic of Korea). We maintained the cells at 37 °C in a humidified atmosphere of 5% CO_2_.

### 4.4. Cell Viability Assay

We evaluated the cytotoxicity of *R. rugosa* Thunb. flower bud extracts in 3T3-L1 preadipocyte cells using the MTT reduction assay. We seeded undifferentiated 3T3-L1 cells in 96-well plates at a density of 1 × 10^5^ cells/mL (100 µL per well) and incubated them for 24 h. We diluted the extract in serum-free medium and applied it to the cells for an additional 24 h. After treatment, we removed the medium and added 100 µL of MTT solution (0.5 mg/mL) into each well. We incubated the cells for 4 h at 37 °C, solubilized the resulting formazan crystals in 100 µL of dimethyl sulfoxide, and measured the absorbance at 570 nm using a UV/Vis spectrophotometer (Opiwen 2120UV plus, Mecasys Co., Ltd., Yuseong-Gu, Republic of Korea). We calculated cell viability relative to the untreated control group.

### 4.5. Lipid Accumulation in 3T3-L1 Cells

We assessed how *R. rugosa* Thunb. flower bud extracts affect lipid accumulation via Oil Red O staining. We seeded 3T3-L1 preadipocyte cells in 6-well plates at a density of 5 × 10^5^ cells/well and cultured them until reaching confluence. Two days post-confluence, we initiated differentiation with DMEM containing 0.5 mM IBMX, 0.5 µM dexamethasone, 10 µg/mL insulin, and 10% fetal bovine serum (FBS) for 2 days. Next, we replaced the medium with DMEM containing 10 µg/mL insulin and 10% FBS for an additional 2 days, followed by DMEM with 10% FBS for 4 days to complete differentiation. In total, the adipocyte differentiation period lasted 8 days. During the differentiation period, NG-RR-T1F extracts were administered at concentrations of 50, 75, and 100 µg/mL with every medium change to ensure consistent exposure throughout the adipogenic process. At the end of differentiation, we washed the cells twice with PBS and fixed them with 10% formalin for 1 h, followed by washing with distilled water. We stained the cells with 60% Oil Red O solution for 1 h and then washed, dried, and eluted them with 100% isopropanol. We measured absorbances at 520 nm to quantify intracellular lipid accumulation.

### 4.6. Triglyceride Content in 3T3-L1 Cells

We measured triglyceride levels using the EZ-Triglyceride Quantification Assay Kit. We induced differentiation as described above, applying NG-RR-T1F at concentrations of 50, 75, and 100 µg/mL throughout the process. At the end of differentiation, we washed the cells twice with PBS, lysed them using NP-40, heated the lysates at 100 °C to solubilize intracellular triglycerides, and centrifuged them at 13,000 rpm for 2 min to remove the insoluble debris. We transferred 50 µL of the supernatant and standard into 96-well plates with 2 µL of lipase and incubated them at room temperature for 20 min. Finally, we supplemented the samples with 46 L of assay buffer, 2 µL of enzyme mix, and 2 µL of probe, incubated the mixtures for 30 min, and measured absorbances at 570 nm.

### 4.7. Quantitative Real-Time PCR in 3T3-L1 Cells

Differentiated 3T3-L1 adipocytes were treated with NG-RR-T1F extract at concentrations of 50, 75, and 100 µg/mL and incubated for 24 h. Total RNA was extracted using QIAzol^®^ Lysis Reagent (Qiagen, Hilden, Germany) according to the manufacturer’s instructions. The RNA concentration was quantified, and 2 µg of total RNA was used to synthesize complementary DNA (cDNA) with the amfiRivert™ cDNA Synthesis Platinum Master Mix (GenDEPOT, Katy, TX, USA). Quantitative real-time reverse transcription PCR (RT-qPCR) was conducted using a LightCycler^®^ 480 Instrument II (Roche Diagnostics, Basel, Switzerland). The primer sequences used are listed in Table 2. RT-qPCR was performed using the EzAmp™ qPCR 2X Master Mix (Roche Diagnostics, Basel, Switzerland). Gene expression levels were calculated using the 2^−ΔΔCt^ method. The thermal cycling conditions were as follows: initial denaturation at 95 °C for 5 min, followed by 40 cycles of 95 °C for 15 s, 58 °C for 15 s, and 72 °C for 30 s. Gene expression was normalized to glyceraldehyde-3-phosphate dehydrogenase (GAPDH) as an internal control, and fold changes were calculated relative to the reference gene.

### 4.8. Western Blot Analysis in 3T3-L1 Cells

We treated fully differentiated 3T3-L1 adipocytes with NG-RR-T1F at 50, 75, and 100 µg/mL for 24 h, harvested the cells using a cell scraper, centrifuged them at 5000 rpm for 10 min, and lysed them in radioimmunoprecipitation assay (RIPA) buffer. We quantified protein concentrations using the Bradford assay, mixing equal amounts of protein with β-mercaptoethanol-containing sodium dodecyl sulfate (SDS) sample buffer (at a ratio of 3:1) and heating the samples at 100 °C for 10 min. We separated the samples via SDS-polyacrylamide gel electrophoresis, transferred them onto polyvinylidene fluoride membranes (0.45 µm, Thermo, Rockford, IL, USA), and blocked the membranes with Tris-buffered saline (TBS) (containing 0.1% Tween-20 and 5% skim milk) for 2 h, followed by overnight incubation with the primary antibodies. After washing with TBS-T, we incubated the membranes with HRP-conjugated secondary antibodies (1:1000, Cell Signaling Technology, Danvers, MA, USA) for 1 h. We visualized the protein bands using an enhanced chemiluminescence system and quantified the results using an image analyzer (LAS4000, Fujifilm, Tokyo, Japan) and ImageJ software (Version 1.53, NIH, Bethesda, MD, USA).

### 4.9. Animal Experiments

To evaluate the anti-obesity effects of the NG-RR-T1F extract, we used six-week-old male Sprague Dawley rats obtained from Daehan Biolink Co. (Chungbuk, Republic of Korea). We conducted all animal procedures in accordance with the recommendations of the Institutional Animal Care and Use Committee of Chaon Co., Ltd. (approval number: CE23524). During the experimental period, we housed the animals in a controlled environment maintained at 23 ± 3 °C, with 50 ± 20% of relative humidity, a ventilation rate of 10–15 air changes per hour, and a 12 h light/dark cycle (150–200 lux). We acclimated the animals for one week with free access to standard rodent chow and water.

Following acclimatization, we randomly divided the animals into six groups (*n* = 8 per group) as follows: G1, normal diet (ND); G2, high-fat diet (HFD); G3, HFD + hydroxycitric acid 150 mg/kg body weight (BW); G4, HFD + NG-RR-T1F 50 mg/kg BW; G5, HFD + NG-RR-T1F 75 mg/kg BW; and G6, HFD + NG-RR-T1F 100 mg/kg BW.

All treatments were administered once daily by oral gavage for 12 consecutive weeks throughout the experimental period. The ND group received AIN-76 diet, while groups G2–6 received a 60% kcal HFD (D12492, Research Diets, New Brunswick, NJ, USA). We suspended the NG-RR-T1F extract in 0.1% methylcellulose prior to administration. We measured body weight twice weekly at the same time of the day and removed the food one hour prior to weighing to minimize measurement errors associated with recent food intake.

One day prior to necropsy, we measured body composition (i.e., fat, lean, and free water masses) using Echo-magnetic resonance imaging.

### 4.10. Necropsy

At the end of the experimental period, we anesthetized the animals using 2% isoflurane, collected their blood via the abdominal aorta, transferred the blood samples into SST tubes (367957, BD SST II Vacutainer, BD, Franklin Lakes, NJ, USA), and centrifuged them at 5000 rpm for 10 min to obtain the serum. We stored the collected serum at −80 °C in pre-labeled tubes. After blood collection, we transected the abdominal aorta and vein to complete exsanguination and then carefully excised, weighed, and fixed the liver as well as the retroperitoneal, epididymal, and visceral fat tissues in 10% neutral-buffered formalin.

### 4.11. Serum Biochemical Analysis

We measured biochemical parameters in the collected serum using an automated blood chemistry analyzer (AU680, Beckman Coulter, Brea, CA, USA). The analyzed markers included free fatty acids, total cholesterol, LDL-C, HDL-C, triglycerides, ALP, AST, ALT, and GGT.

### 4.12. Histological Analysis

We processed the previously fixed liver and retroperitoneal fat tissues using a tissue processor (Tissue-Tek VIP 5 Jr, SAKURA Finetek, Tokyo, Japan), followed by paraffin embedding using an embedding system (LEICA EG1150H, LEICA, Wetzlar, Germany). We sectioned the paraffin blocks at a thickness of 5 µm using a rotary microtome (HM 340E, Thermo Scientific, Waltham, MA, USA). Next, we deparaffinized, rehydrated, and stained the tissue sections with hematoxylin and eosin. We analyzed the microscopic images using the DIXI eXcope image analysis system. In the liver tissue, we measured the LD area to assess hepatic steatosis (% area). In the retroperitoneal fat tissue, we measured the average diameter of at least 10 adipocytes per sample to determine adipocyte size.

### 4.13. Quantitative Real-Time PCR of Epididymal Adipose Tissue

A total of 100 mg of epididymal fat tissue collected during necropsy was washed and dehydrated, and total RNA was extracted using the QIAzol^®^ Lysis Reagent (Qiagen, Hilden, Germany) in accordance with the manufacturer’s instructions. We performed quantitative real-time PCR using the same protocol described for the cell-based experiments. The primer sequences used are listed in Table 2.

### 4.14. Western Blot Analysis of Epididymal Adipose Tissue

We washed and blotted dry the epididymal fat tissue samples collected during necropsy and then homogenized them using 1 g of tissue per 1 mL of RIPA buffer (composition: 20 mM Tris-HCl [pH 7.5], 150 mM NaCl, 1 mM Na_2_EDTA, 1 mM EGTA, 1% NP-40, 1% sodium deoxycholate, 2.5 mM sodium pyrophosphate, 1 mM β-glycerophosphate, and 1 mM Na_3_VO_4_). We homogenized the samples at 4 °C using a tissue homogenizer (HS30E, DAIHAN, Seoul, Republic of Korea), centrifuged the homogenates at 10,000× *g* for 10 min, and collected the supernatant. We performed Western blotting using the same protocol described for the cell-based experiments.

### 4.15. Statistical Analysis

We expressed all data as the mean ± SD and performed statistical analysis using GraphPad Prism software (version 8.0.2; GraphPad Software, San Diego, CA, USA). We used one-way analysis of variance followed by Duncan’s multiple range test for post hoc analysis. We considered *p*-values < 0.05 statistically significant.

## 5. Conclusions

In this study, we confirmed that *R. rugosa* flower bud extract regulates fat metabolism by fat accumulation, lipolysis, lipogenesis, and energy expenditure in 3T3-L1 adipocytes and that it has effects on body weight, organ weight, fat mass, tissue fat accumulation, and size regulation in SD rats with high-fat diet-induced obesity. Based on this study, we confirmed that NG-RR-T1F, an *R. rugosa* flower bud extract, has a body fat reduction effect. It is decided that it can stimulate fat metabolism without side effects by enhancing intracellular lipolysis, inhibiting fat synthesis, and increasing energy expenditure. It is anticipated that NG-RR-T1F can be developed as a health functional food for body fat reduction through future studies involving the standardization of raw materials, safety evaluations, and GLP-compliant toxicity testing.

## Figures and Tables

**Table 1:** HPLC conditions for the quantitative analysis of phenolic acid composition.

Items	Conditions			
Instrument	SHIMADZU LC-20A			
Detector	SHIMADZU PDA Detector (SPD-M20A, wave length at 254 nm)			
Column	ZORBAX ECLIPSE XDB-C18 (150 mm × 4.6 mm, 3.5 µm)			
Mobile phase	(A) 0.2% formic acid in water (B) 0.2% formic acid in acetonitrile			
	Time (min.)	Flow rate (mL/min)	(A)	(B)
	0.00	1.00	95	5
	50.00	1.00	0	100
	55.00	1.00	0	100
	55.01	1.00	95	5
	60.00	1.00	95	5
Run time	60 min			
Flow rate	1.0 mL/min			
Injection volume	10 µL			
Column temperature	30 °C			

**Table 2:** List of primers used for real-time PCR.

Gene	Primer	*Mus musculus*	*Rattus norvegicus*
		Sequences (5′ → 3′)	Sequences (5′ → 3′)
ATGL	Forward	CATTCTCAGGCGAGAGTGACAT	AGACTGTCTGAGCAGGTGGA
	Reverse	GACGCGAAGCTCGTGGAT	AGTAGCTGACGCTGGCAT
G0S2	Forward	AGAAGAACGCCAAAGCCAGT	AGCATGCCTCTTAAGGCTGG
	Reverse	AGCTCCTGCACACTTTCCAT	GGATTCGGTGGCACCTTGAA
PLIN1A	Forward	GCGTCTGCCTTACCTAGCT	GAGTCACAACCCCACGATGT
	Reverse	TGGGCTTCTTTGGTGCTGTT	CGAGAGAGGAAAGAGTCGAC
ACC	Forward	GAATCTCCTGGTGACAATGCTTATT	GCCTCCAACCTCAACCACTA
	Reverse	GGTCTTGCTGAGTTGGGTTAGCT	TCGCAGAAGCAGCCCATTA
FAS	Forward	CTGAGATCCCAGCACTTCTTGA	GAGCCGCCGACCAGTATAAA
	Reverse	GCCTCCGAAGCCAAATGAG	GCACAGACACCTTCCCATCA
CPT-2	Forward	TCCGCTTTGTTCCTTCCTCT	GCCCAGCCTCCATCTTTACT
	Reverse	TCACGACTGGGTTTGGGTAT	AGCGCAGAGCATACAAGTGT
UCP-1	Forward	ACGGGGACCTACAATGCTT	TGGCGTGGCGGTATTCATT
	Reverse	GCAAAACCCGGCAACAAGA	GAGTCGTCCCTTTCCACAGT
PGC-1α	Forward	TGAAGAGCGCCGTGTGATT	TGAACTACGGGATGGCAACT
	Reverse	AAGAGCAGCGAAAGCGTCA	AAGAGCAAGAAGGCGACACA
AMPK1α	Forward	GTCAAAGCCGACCCAATGATA	CACTGGATGCACTCAACACAAC
	Reverse	CGTACACGCAAATAATAGGGGTT	TCACTACCTTCCATTCAAAGTCC
GAPDH	Forward	CATGGCCTTCCGTGTTCCTA	CTGGAGAAACCTGCCAAGTATG
	Reverse	GCGGCACGTCAGATCCA	GGTGGAAGAATGGGAGTTGCT

## Data Availability

All data generated or analyzed during this study are included in this published article.

## Abbreviations

The following abbreviations are used in this manuscript:

IR	Insulin resistance
FFA	Free fatty acid
ROS	Reactive oxygen species
AMPK	5′-adenosine monophosphate-activated protein kinase (AMPK)
PPARγ	Peroxisome proliferator-activated receptor gamma ()
C/EBPα	CCAAT/enhancer-binding protein alpha
SREBP-1c	Sterol regulatory element binding protein 1c
LD	Lipid droplet
ATGL	Adipose triglyceride lipase
HSL	Hormone-sensitive lipase
MGL	Monoglyceride lipase
PKA	Protein kinase A
ACC	Acetyl-CoA carboxylase
FAS	Fatty acid synthase
HPLC	High-performance liquid chromatography
MTT	3-(4,5-dimethylthiazol-2-yl)-2,5-diphenyl tetrazolium bromide
CPT	Carnitine palmitoyltransferase
HFD	High-fat diet
ND	Normal diet
LDL-C	Low-density lipoprotein cholesterol
HDL-C	High-density lipoprotein cholesterol
AST	Aspartate aminotransferase
ALT	Alanine aminotransferase
ALP	Alkaline phosphatase
GGT	Gamma-glutamyltranspeptidase
CGI-58	Comparative gene identification-58
G0S2	G0/G1 Switch 2
PGC-1α	PPARγ coactivator 1 alpha
DMEM	Dulbecco’s Modified Eagle Medium
FBS	Fetal bovine serum
RIPA	Radioimmunoprecipitation assay
SDS	Sodium dodecyl sulfate
TBS	Tris-buffered saline
TBS-T	Tris-buffered saline containing Tween-20
