# Correction: Kim et al. Anti-Obesity Effects of *Rosa rugosa* Thunb. Flower Bud Extracts on Lipid Metabolism Regulation in 3T3-L1 Adipocytes and Sprague Dawley Rats. *Int. J. Mol. Sci.* 2025, *26*, 6963

**DOI:** 10.3390/ijms26209942

**Published:** 2025-10-13

**Authors:** Jung Min Kim, Kyoung Kon Kim, Hye Rim Lee, Jae Cheon Im, Tae Woo Kim

**Affiliations:** Newgen Healthcare Co., Ltd., Chuncheon-si 24232, Republic of Korea; jmkim@newgenhc.co.kr (J.M.K.); gon87@newgenhc.co.kr (K.K.K.); hyerim0708@newgenhc.co.kr (H.R.L.); ccyim@newgenhc.co.kr (J.C.I.)

The authors wish to make the following correction to this paper [1]. The authors state that the scientific conclusions are unaffected. This correction was approved by the Academic Editor. The original publication has also been updated.

Error in Figure

In the original publication, there was a mistake in Figure 6a as published. The statistical significance marking for group G6 was incorrectly shown as “###”. The corrected Figure 6a appears below, in which the significance marking for group G6 has been changed to “#”.

Results, Section 2.9 (Paragraph 3)

There was an error in the original publication. In Results, Section 2.9, Paragraph 3, several numerical values were inconsistent with those shown in Figure 6.

Corrected:

Liver weight significantly increased in G2 (13.11 g) compared to G1 (9.38 g). NG-RR-T1F administration significantly reduced liver weight in all treatment groups as follows: G4 (11.07 g), G5 (11.19 g), and G6 (11.14 g) (Figure 6a). Retroperitoneal fat weight increased significantly in G2 (21.67 g vs. 6.71 g in G1) but significantly decreased in G4 (14.07 g), G5 (14.88 g), and G6 (14.26 g) (Figure 6b). Moreover, epididymal fat weight significantly increased in the HFD group (18.55 vs. 7.28 g in G1). Although we observed reductions in the NG-RR-T1F groups, i.e., G4 (15.02 g), G5 (15.01 g), and G6 (12.80 g), only the G6 group showed a statistically significant decrease, whereas G4 and G5 did not exhibit significant differences (Figure 6c). Visceral fat weight significantly increased in G2 (13.04 vs. 4.06 g in G1) and significantly decreased in G4 (7.46 g), G5 (7.63 g), and G6 (6.85 g) (Figure 6d).

Results, Section 2.10 (Paragraphs 2–3)

There was an error in the original publication. In Results, Section 2.10, Paragraphs 2–3, several fat mass values in the text were inconsistent with those shown in Figure 7.

Corrected:

Fat mass was significantly higher in the HFD (183.27 g) compared to the ND (84.02 g) group. NG-RR-T1F treatment significantly reduced fat mass to 143.30 g (G4), 151.04 g (G5), and 134.57 g (G6) (Figure 7a). Lean mass did not significantly differ among the groups: G2 (386.69 g), G1 (349.30 g), G4 (390.64 g), G5 (370.72 g), and G6 (383.04 g) (Figure 7b). Bone mineral content slightly increased in G2 (11.15 g) compared to G1 (9.77 g), but NG-RR-T1F exhibited no significant effect: G4 (10.63 g), G5 (10.95 g), and G6 (11.22 g) (Figure 7c).

Results, Section 2.11 (Paragraph 1)

There was an error in the original publication. In Results, Section 2.11, Paragraph 1, the order of lipid parameters described in the text did not match that shown in Figure 8.

Corrected:

Total cholesterol was significantly elevated in G2 (97.25 mg/dL vs. 73.25 mg/dL in G1). G4 (76.50 mg/dL) displayed a significant reduction compared to G2, while G5 (84.25 mg/dL) and G6 (90.38 mg/dL) did not (Figure 8a). Moreover, serum triglyceride levels significantly increased in the HFD group (69.25 vs. 42.63 mg/dL in G1). Among the treatment groups, only G6 (52.13 mg/dL) yielded a statistically significant reduction compared to G2 (Figure 8b). High-density lipoprotein cholesterol (HDL-C) levels did not differ significantly between G2 (25.50 mg/dL) and G1 (28.38 mg/dL), nor among NG-RR-T1F-treated groups (G4, 5, and 6: 24.88, 28.00, and 28.25 mg/dL, respectively) (Figure 8c). Low-density lipo-protein cholesterol (LDL-C) levels were not significantly different between G2 and G1 (9.00 and 7.13 mg/dL, respectively). However, G4 (6.13 mg/dL) exhibited a significant reduction compared to G2 (Figure 8d).

## Figures and Tables

**Figure 6:**
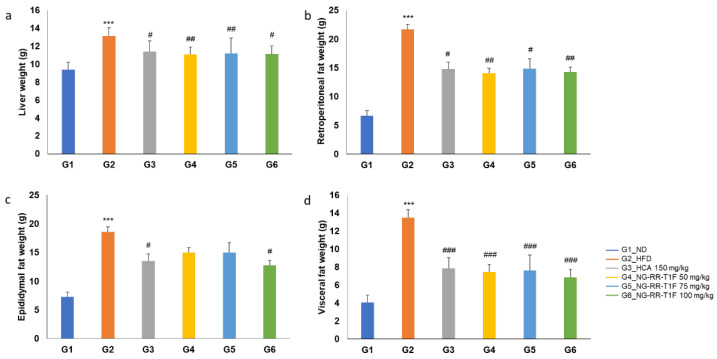
Effects of NG-RR-T1F on absolute organ weights in HFD-induced obese rats. (**a**) Liver weight, (**b**) retroperitoneal fat weight, (**c**) epididymal fat weight, and (**d**) visceral fat weight. The values are expressed as the mean ± SD (*n* = 8). *** *p* < 0.001 vs. G1_ND; # *p* < 0.05, ## *p* < 0.01, and ### *p* < 0.001 vs. G2_HFD.
